# Presymptomatic viral shedding and infective ability of SARS-CoV-2; a case report

**DOI:** 10.1016/j.heliyon.2021.e06328

**Published:** 2021-02-19

**Authors:** Karolina Nissen, Marie Hagbom, Janina Krambrich, Dario Akaberi, Sumit Sharma, Jiaxin Ling, Tove Hoffman, Lennart Svensson, Kåre Bondeson, Erik Salaneck

**Affiliations:** a Dept of Medical Sciences, Uppsala University, Uppsala, Sweden; b Dept of Biomedical and Clinical Sciences, Linköping University, Linköping, Sweden; c Dept of Medical Biochemistry and Microbiology, Uppsala University, Uppsala, Sweden; d Dept of Medicine, Karolinska Institute, Stockholm, Sweden

**Keywords:** SARS-CoV-2, COVID-19, Presymptomatic transmission, Cell culture, Infectivity, Antibody, Health care workers, Epidemiology, Coronavirus

## Abstract

Possible pre- or asymptomatic transmission has been reported, both from SARS-CoV and from MERS-CoV outbreaks, although this appears to be uncommon. In contrast, during the COVID-19 pandemic, an increasing number of studies and case reports indicate that pre- or asymptomatic transmission of SARS-CoV-2 is not only possible but also occurs frequently. We report repeated rRT-PCR detection of SARS-CoV-2 in a health care worker and demonstrate infective ability up to three days prior to mild COVID-19 symptoms. rRT-PCR indicated high viral levels approximately three days after exposure. Viral samples collected one and three days prior to symptoms exhibited infectivity on Vero E6 cells, confirmed by detection of double-stranded RNA by immunofluorescence, assessment of cytopathic effect (CPE) and rRT-PCR. SARS-CoV-2 specific IgM and IgG antibodies were detected by day 9 and 15, respectively, after symptom onset. We propose that this provides evidence for potential early presymptomatic transmission of SARS-CoV-2 and that infectivity may be manifest shortly after exposure.

## 1 Introduction

SARS-CoV-2 causes coronavirus disease 2019 (COVID-19) which has rapidly spread globally since December 2019, resulting in a pandemic declared by the World Health Organization (WHO). Reports of possible pre- or asymptomatic transmission in two other emerging coronaviruses have been published, both from the outbreak of SARS-CoV in 2003 and from outbreaks of MERS-CoV, although it seems to be uncommon, possibly due to a low viral load at the time of symptom onset [1, 2, 3, 4, 5]. In contrast, during the current SARS-CoV-2 pandemic, an increasing number of studies and case reports indicate that pre- or asymptomatic transmission is not only possible but also seems to occur more frequently than with SARS CoV and MERS-CoV [6, 7, 8, 9, 10, 11, 12, 13, 14]. Although the main transmission routes for SARS-CoV-2 are considered to be droplets or direct contact with symptomatic individuals, the rapid, global spread of COVID-19 warrants further investigations into other transmission modes as well as the potential of contagiousness prior to symptom onset [11, 12, 15].

In this report, we present a case of presymptomatic viral shedding and infective ability of SARS-CoV-2 in a health-care worker (HCW) and following serological response in a mild case of COVID-19.

## 2 Case report

On April 2, 2020, a previously healthy 36-year-old woman and HCW at a nursing home, was exposed, without proper personal protective equipment (PPE), to a patient with respiratory symptoms who was determined to have COVID-19 by real-time reverse-transcriptase–polymerase-chain-reaction (rRT-PCR) diagnosis. Due to exposition she was referred for COVID-19 screening at Uppsala University Hospital. On April 5, 2020, while still not exhibiting any symptoms, a nasopharyngeal (NP) and oropharyngeal (OP) swab for SARS-CoV-2 was taken and found to be positive by rRT-PCR. Since the patient still did not reveal any symptoms, the test was repeated on April 7, to verify the results, and once again turned out strongly positive for SARS-CoV-2 by rRT-PCR. Upon second investigation no symptoms were identified, and body temperature was measured to 37.0 °C.

On April 8, the patient developed a temperature of 38 °C, nasal congestion and mild sinusitis symptoms. By April 10, the fever had subsided, but the nasal congestion continued until April 12, 2020. On April 13, 2020, she had fully recovered. On April 14, a further PCR-analysis for SARS-CoV-2 was performed from an OP swab and saliva. The saliva sample was negative, but the OP swab remained positive. On April 17, 9 days after onset of mild COVID-19 symptoms, the patient tested negative for SARS-CoV-2 by PCR-analysis (OP swab and saliva) and isolation measures were lifted. Rapid serological testing was repeatedly performed at all follow-ups revealing development of IgM at day 9, and IgG at day 15 after symptom onset.

## 3 Material and methods

### 3.1 Specimen collection

Specimens were obtained at a polyclinical testing facility for COVID-19 at Uppsala University Hospital. NP and OP swabs (eSwab, Copan Italia SpA, Italy) were acquired; the two swabs were inserted into the same sterile tube containing 1 ml of viral transport medium. Saliva was collected in a sterile container. Specimens were stored at 4 °C until transport.

Capillary blood (20 μl) samples were collected, analyzed and directly assessed, using a commercial rapid test for detection of SARS-CoV-2-specific IgM and IgG (Zhejiang Orient Gene Biotech, Zhejiang, China) according to the manufacturer's instruction [16].

### 3.2 rRT-PCR clinical specimens

Duplex rRT-PCR targeting the SARS-CoV-2 Envelope small membrane protein (E) and Nucleocapsid (N) genes was performed according to Corman et al. utilizing standard diagnostic routines at Uppsala University Hospital [17, 18].

### 3.3 Cell culture

Vero E6 cells, green monkey kidney cells (ATCC® CRL-1586™) were cultured at 37 °C and 5% CO_2_, in Dulbecco's Modified Eagle's Medium (DMEM) (Gibco, Code: 13345364), supplemented with 10% fetal calf serum (FCS). Cell cultures and infection was performed in two different bio-safety level 3 (BSL-3) laboratories. For details see supplementary materials.

### 3.4 Infection of Vero E6 cells for CPE and rRT-PCR analyses

The two laboratories used different approaches for detection of infectious virus particles from the clinical samples. Virus infection was either performed in a T25 flask, checked for CPE daily and increase in viral load determined 96 h post infection, or, infection was done in a 6 well plate and observed for CPE and increase in viral load determined every 24 h for 4 days (for details see supplement). For immunofluorescence, DMEM washed cells were infected with 150 μl of patient samples; 4.71×10^6^ and 9.18×10^5^ genome copies/ml for first and second sample respectively. Cell cultures followed by CPE-observation and rRT-PCR were infected with 600 μl of medium from patient specimens. In both cultures, cells were infected for 2 h, washed, and then incubated in DMEM containing 2% FCS at 37 °C, % CO_2,_ until harvested after 92 h post-infection (hpi) (immunofluorescence), or CPE observed and evaluated by rRT-PCR every 24 h up to 92 hpi.

### 3.5 Anti-dsRNA immunofluorescence

For immunofluorescence, 500 μl of the Vero E6 cell suspension (concentration 0.5×10^6^ cells) in DMEM containing 2% FBS and gentamycin (100 μg/mL) was added to each well of a 24-well plate. Patient samples were serially diluted two-fold in DMEM and added to each well. The starting dilution was 2.35×10^5^ and 4.59×10^4^ genome copies for patient sample 1 and 2, respectively. The plate was then incubated at 37 °C with 5% CO_2_ for 48 h.

Infected cells were fixed with 4% formaldehyde in phosphate buffer saline (PBS) for 1 h, and permeabilized with 0.5% triton-X in PBS for 10 min. Mouse-anti-dsRNA antibody (Scions, Code: J2) was added and incubated for 90 min, followed by incubation with goat-anti-mouse Alexa^488^ (Jackson Immuno research, Code: 115-545-003, USA) for 1 h. Cells were incubated with DAPI nuclear stain (5 μg/mL, Invitrogen, Code: D1301) for 2 min, and infected cells visualized. All incubations were performed at room temperature and all washes with PBS.

### 3.6 RNA extraction and real time PCR

RNA was extracted from infected cell culture supernatant with QIAamp Viral RNA Mini Kit (Qiagen, Hilden, Germany) or using TRIzol® (ThermoFisher, USA) according to manufacturer's protocols. In the two different cell culture experiments, reverse transcription and subsequent rRT-PCR was done according to the following: for immunofuorescence reverse transcription was performed with iScript™ cDNA Synthesis Kit (Biorad, Solna, Sweden) and the subsequent rRT-PCR using iTaq Universal Probes Supermix (Biorad, Solna, Sweden), primers (RdRp_SARSr-F and RdRp_SARSr-R) and probe (RdRp_SARSr-P2) targeting the RdRp gene as described by Corman et al (16). Alternatively, for the cell cultures examined for cytopathic effect, SARS-CoV-2 genes E and N were amplified by an rRT-PCR assay using the SuperScript III OneStep rRT-PCR System with Platinum® Taq DNA Polymerase kit (Invitrogen, Cat. No. 12574026) according to manufacturer's instructions and cycling conditions according to Corman et al, Wang et al [17, 19].

Written informed consent was obtained from the patient to publish the details included in this case report. Case reports do not require ethical approval in Sweden.

## 4 Results

### 4.1 Diagnostic rRT-PCR

The first specimen (NP and OP pooled) for SARS-CoV-2 obtained 3 days prior to symptom onset, was positive for both the E- and the N-genes (cycle threshold (Ct) values NP + OP E-gene: 17.23 and NP + throat N-gene: 17.80). After 2 days (1 day prior to symptoms) the results were again positive for both genes (Ct values NP + OP E-gene: 16.97 and NP + OP N-gene: 16.75). 2 days after symptom resolution, a third throat swab and a saliva specimen were collected. The saliva sample was negative, but the OP swab positive (E-gene: 33.76 and N-gene: 32.63). 9 days after symptom onset, viral RNA was not detected (OP swab and saliva).

### 4.2 Infection of Vero E6 cells and immunofluorescence with anti-ds-RNA

**Figure 1:**
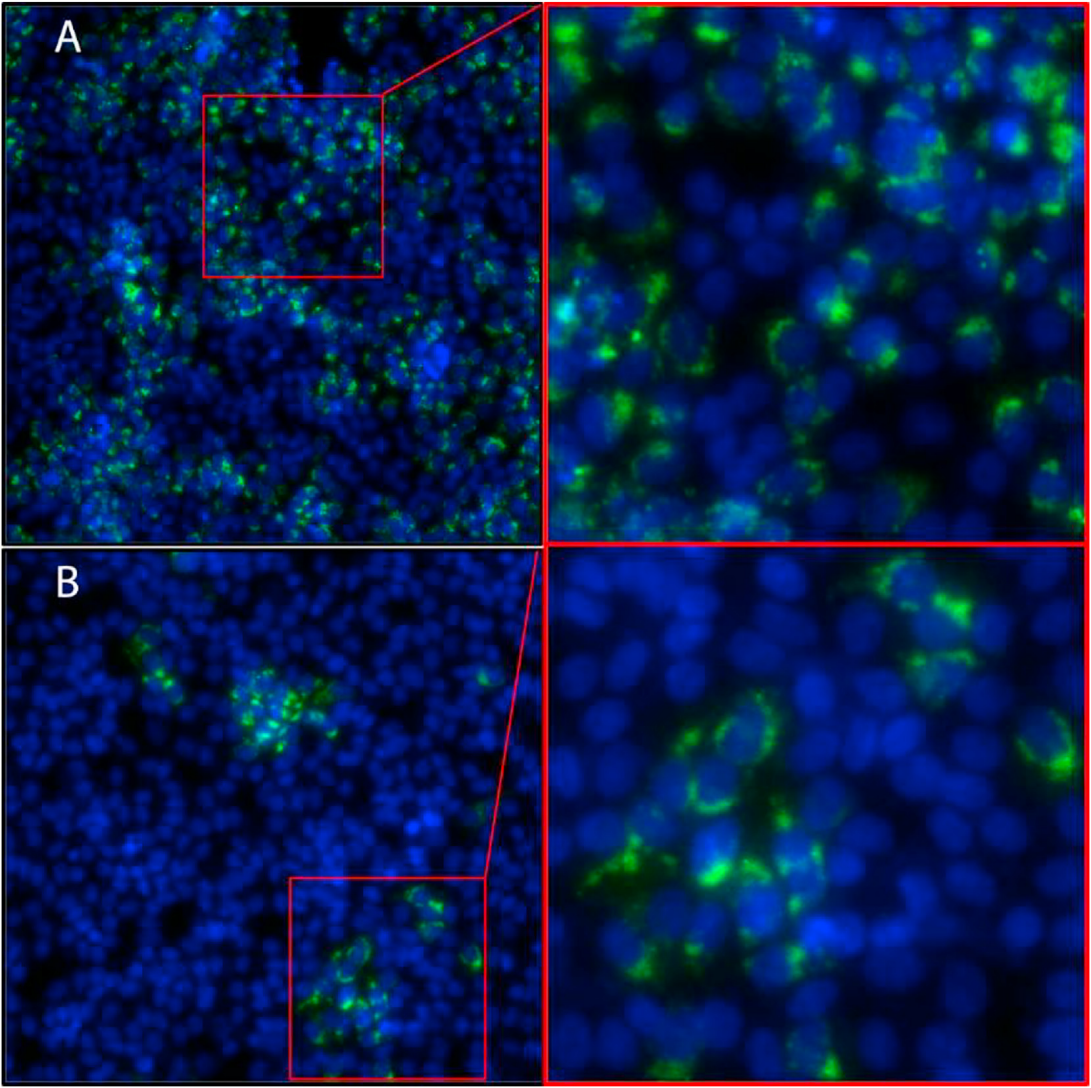
Detection of replicating SARS-Cov-2 with mouse-anti dsRNA (green) in Vero E6 infected cells 48 h post inoculation with nasopharyngeal samples from an asymptomatic individual. Both clinical samples analyzed, (A) sample 1 (3 days prior to symptoms) and (B) sample 2 (1 day prior to symptoms) were found to replicate in Vero cells. The images were captured with 20x objective and the left picture in A and B is a magnification of the red area.

**Figure 2:**
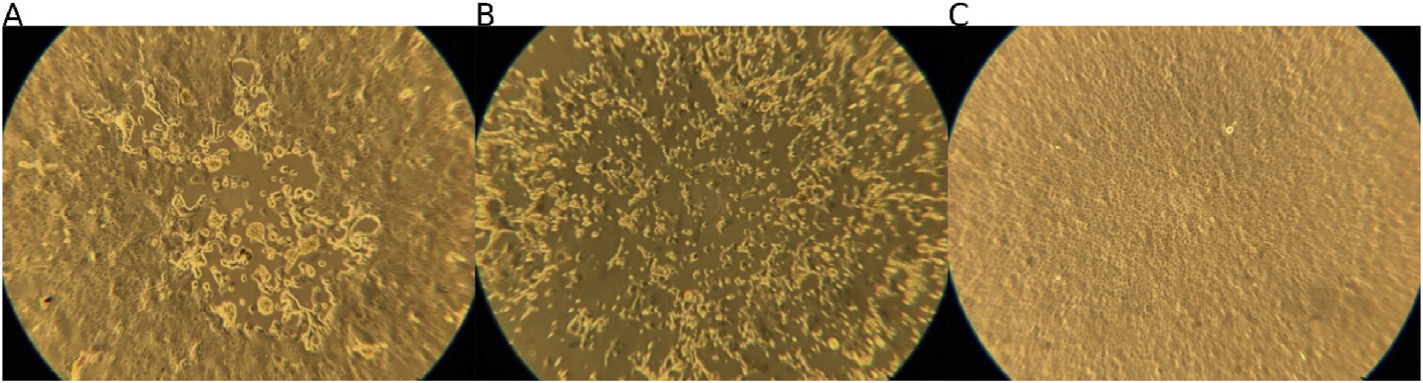
Cytopathic effect observed in Vero-E6 cells at 48 (A) and 72 (B) hours post inoculation and (C) an uninfected control. Cells in (A) and (B) were inoculated with the patient sample collected 72 h prior to onset of symptoms.

Infection experiments were performed in two different BSL-3 laboratories. Significant virus replication was observed by immunofluorescence detection of dsRNA as seen in Figure 1. Additionally, real-time PCR performed from cell lysates indicated viral replication. Four days post infection, 1.36×10^11^ and 2.02×10^11^ genome copies/ml were detected in cell lysates for patient samples 1 and 2 respectively. A 2.9×10^4^ and 2.2 × 10^5^-fold increase in the genome copy number was detected compared to that in the initial inoculum for patient sample 1 (4.71×10^6^ genome copies/ml) and patient sample 2 (9.18×10^5^ genome copies/ml) respectively. In the parallel cell culture experiment, cytopathic effect (CPE) was followed at 24-hour intervals, up to 92 h. 70% CPE was observed for both samples; 48 hpi for patient sample 1 (3 days before symptoms) and 72 hpi for patient sample 2 (1 day before symptoms) (see Figure 2). rRT-PCR performed on culture supernatant at the same intervals exhibited decreasing Ct-values, further indicating viral replication (Table 1). Thus, observed CPE, immunofluorescence and rRT-PCR determined that the SARS-Cov-2 virus from both samples retrieved from the asymptomatic patient, 3 days and 1 day prior to symptom onset, were infectious in two separate experiments.

**Table 1:** rRT-PCR detection and CPE for patient samples and the viral isolation. Patient sample 1 was acquired 3 days prior to symptom onset and sample 2, 1 day prior.

Patient sample	Passage	Timepoint (hpi)	E gene Ct value	N gene Ct value
1	1	0	34.03	42.35
1	1	48^a^	29.79	37.49
1	1	72	18.18	21.08
1	2∗	24^a^	24.16	29.11
1	2∗	48	15.25	17.57
2	1	0	26.22	27.88
2	1	48	24.18	29.81
2	1	72^a^	18.25	19.70
2	1	96	12.46	14.79

a Timepoint when clear CPE was observed.

∗ 200 μl of the P1 48 h were used to inoculate a T-25 flask.

### 4.3 Rapid test detection of SARS-CoV-2-specific IgM and IgG

During patient follow-up, a commercial rapid test was used for detection of SARS-CoV-2-specific IgM and IgG. On day 1 prior to and day 6 after symptom onset neither IgM nor IgG were detected. 9 days after symptom onset IgM was positive, but IgG negative. 15 days after symptom onset both IgM and IgG were detected.

## 5 Discussion

A HCW with exposure to SARS-CoV-2 was tested twice using standard clinical rRT-PCR diagnostics and exhibited low Ct-values in rRT-PCR analyses, suggesting high viral load, well prior to debut of COVID-19 symptoms. Specimens from the presymptomatic phase exhibited distinct infectious ability when subjected to Vero E6 cell lines. The patient later developed very mild COVID-19 symptoms which lasted only a few days. Virus isolation was not attempted from specimens collected after symptom onset. This was mainly due to the clinical focus on presymptomatic viral shedding in this patient and the potential for viral transmission prior to symptom onset. Studies have shown it is uncommon to detect SARS-CoV-2 with maintained infectious ability more than 9 days after symptom onset [20]. It would however have been valuable to determine the duration of infectious viral shedding in this patient, nevertheless in the clinical setting focus remained on the risk for pre- or asymptomatic transmission. Seroconversion was followed and both IgM and IgG were detected 15 days after symptom onset. The findings in this case report not only indicate extensive presymptomatic viral replication in the upper respiratory tract, which is a feature of SARS-CoV-2 that clearly differs from SARS-CoV and MERS-CoV, but also that these virions possess distinct infective ability several days before symptoms appeared and only three days after exposure [19].

These results support the potential for presymptomatic transmission of SARS-CoV-2, also in cases with subsequently very mild COVID-19 disease. Clusters of COVID-19 emanating from pre- or asymptomatic cases have been described [7, 8, 9, 10, 11, 13]. Since asymptomatic individuals will not transfer virus by coughing, other modes of transmission must be investigated. Plausible transmission routes could be via droplets when talking, indirectly via fomites, or via aerosols when talking or breathing, although the individual importance of these different transmission routes in asymptomatic individuals needs to be further investigated [15, 21].

Furthermore, this should influence guidelines for preventive measures. We suggest one such measure to be increased PCR-screening, especially among HCWs who are at risk of transmitting SARS-CoV-2 to patients that have a high risk of morbidity and mortality due to COVID-19. Increased screening of asymptomatic patients admitted to hospitals and in nursing homes should also be considered. Another important control measure is providing HCWs with appropriate PPE and education, to prevent transmission in health-care settings. We also propose that rapid antibody tests could aid in determining previous exposition, if used correctly [16]. Both the Center for Disease Control and Prevention (USA) as well as the National Health Service (UK) recommend preventive measures that take asymptomatic transmission into consideration, but this is not the case in all countries affected by the pandemic [22, 23].

This case report corroborates recent findings that presymptomatic viral shedding could be one of several factors contributing to the current SARS-CoV-2 pandemic [11, 13, 24, 25]. Also, extensive viral shedding and infective potential may occur shortly after exposure to COVID-19 patients and well prior to symptoms. Although this report describes a single case, there is nothing about this patient indicating an unexpected course of the COVID-19 infection. She was previously healthy with no immunocompromising medications or history of complicated infections. Thus, the detection of infective SARS-CoV-2 from upper respiratory specimens collected several days prior to symptom onset, lends further evidence for this to be considered to be a possible common feature of mild to asymptomatic COVID-19 infections. There are now several ways to detect the presence of SARS-CoV-2, both with PCR and antigen tests [26, 27]. To mitigate the pandemic, early diagnosis is crucial to prevent further transmission. This case report adds further evidence to the increasing data indicating possible risk for pre- or asymptomatic transmission [28, 29]. Thus, extensive screening with PCR or antigen tests in order to detect and isolate contagious patients who have not yet developed symptoms, is strongly advised. This is especially important in the context of HCWs, as the risk and consequences of transmission to vulnerable individuals such as patients in hospitals and residents at nursing homes, are substantial. Consequently, in order to be effective, guidelines concerning preventive measures against the spread of SARS-CoV-2, especially in health-care facilities, need to consider the role of pre- and asymptomatic transmission.

## Declarations

### Author contribution statement

All authors listed have significantly contributed to the investigation, development and writing of this article.

### Funding statement

This research did not receive any specific grant from funding agencies in the public, commercial, or not-for-profit sectors.

### Data availability statement

Data included in article/supplementary material/referenced in article.

### Declaration of interests statement

The authors declare no conflict of interest.

### Additional information

No additional information is available for this paper.

## Appendix A Supplementary data

CR Supplement Nissen et al CR Supplement Nissen et al
