# Supplementary material for: Presymptomatic viral shedding and infective ability of SARS-CoV-2; a case report
Source: Heliyon. 2021 Feb 19;7(2):e06328. doi: 10.1016/j.heliyon.2021.e06328 (PMC7894094; doi:10.1016/j.heliyon.2021.e06328)
Supplement: CR Supplement Nissen et al [file mmc1.docx]

**Supplement**

**Presymptomatic viral shedding and infective ability of SARS-CoV-2**

**Karolina Nissen MD^1^, Marie Hagbom^2^, Janina Krambrich**^3^**, Dario Akaberi**^3^**, Sumit Sharma^2^, Jiaxin Ling**^3^**, Tove Hoffman**^3^**, Lennart Svensson PhD^2,4^, Kåre Bondeson MD PhD^1^, Erik Salaneck MD PhD^1^.**

^1^Dept of Medical Sciences, Uppsala University, Uppsala, Sweden

^2^Dept of Biomedical and Clinical Sciences, Linköping University, Linköping, Sweden

^3^Dept of Medical Biochemistry and Microbiology, Uppsala University, Uppsala, Sweden

^4^Dept of Medicine, Karolinska Institute, Stockholm, Sweden

**Cell cultures examined for CPE**

The virus was isolated from two samples collected 72h and 24h prior to onset of symptoms as follows.Vero-E6 cells grown in DMEM (Gibco, 41966) supplemented with 10 % FBS (Gibco, USA) and 1x Penicillin-Streptomycin (Sigma-Aldrich, PA333) were harvested, resuspended in the same cell media and seeded into a 6-wells plate at a cell density of 6x10^5^/well (final volume 2ml/well). After 24h incubation (37°C, 5% CO2) the cell media was substituted with 2ml of DMEM supplemented with 2% FBS, 1x Penicillin-Streptomycin. 600µl of media from the two samples were used to inoculate two different wells (a well for each sample). Infected cells were checked at 24, 48,72, and 96 hours post infection for cytopathic effect and compared to uninfected controls. A total of 50 μl of supernatant from each well at 48 hpi, 72 hpi and 96 hpi were collected for real-time RT-PCR detection. All procedures involving live virus were performed in a biosafety level 3 (BSL-3) laboratory.

Virus isolation was attempted to assess the presence of live SARS-CoV-2 virus in the nasopharyngeal samples acquired 72 and 48 hours before the patient developed symptoms.

Vero-E6 cells seeded into a 6-wells plate were inoculated with 600µl of sample media in which the nasopharyngeal were kept and the development of cytopathic effected (CPE) was monitored at 24 hours intervals for up to four days.

Cells inoculated with the sample taken 72 hours prior to patient onset of symptoms developed evident CPE 48 hours post infection (PI) (Figure 1A). More than 70% of the cell monolayer displayed CPE after 72 hours PI (Figure 1B) at which point the cell supernatant was harvested. Same results could be replicated by transferring 200µl of supernatant collected 72 hours PI into a T-25 flask containing 70% confluent Vero-E6 cells.

The cells inoculated with the sample collected 48 hours prior to onset of symptoms displayed a 24 hours delay with development of evident CPE at 72 hours PI and extended cell monolayer damage/death 96 hours post infection at which point the supernatant was harvested and the experiment concluded.

No CPE was observed in two uninfected control wells throughout the experiment duration (Figure 1C).

Infection kinetic was followed by RT-qPCR performed on supernatant collected at 24, 48, 72 and 96 hours PI time points.

**Cell culture examined by immunofluorescence**

Vero E6 cells, green monkey kidney cells (ATCC® CRL-1586™) were cultured at 37°C and 5% CO2, in Dulbecco´s Modified Eagle´s Medium (DMEM) (Gibco, Code: 13345364), supplemented with 10% fetal bovine serum (FBS) (Gibco, Code: 11550356) and gentamycin (100μg/mL).

**Infection of Vero E6 cells**

For determination of viral replication by PCR, Vero E6 cells grown in T25 culture flasks with confluency of 90-100% were used for infection. The cells were washed twice with serum-free DMEM and infected with 150μl of patient sample containing 4.71×10^6^ and 9.18×10^5^ genome copies/ml for clinical samples 140 and 440 respectively. Two hours post infection, the cells were washed three times with serum-free DMEM, fresh DMEM containing 2% FBS was added, and the flasks were incubated at 37^o^C with 5% CO_2_ until harvested 4 days post-infection for determining increase in genome copies using real time PCR.

For immunofluorescence, Vero E6 cells were trypsinized and pelleted by centrifugation at 290×g for 5 minutes. The pellet was resuspended in DMEM containing 2% FBS and gentamycin (100μg/mL) at a concentration of 0.5×10^6^ cells /ml. 500μl of the cell suspension was added to each well of a 24-well plate, followed by addition of nasopharyngeal samples that were serially diluted two-fold in DMEM. The starting dilution had 2.35×10^5^ and 4.59×10^4^ genome copies for clinical samples 140 and 440 respectively. The plate was then incubated at 37^o^C with 5% CO_2_ for 48 hours before fixation

**Immunofluorescence**

To detect SARS-CoV-2 replication an antibody against dsRNA (Scions, Code: J2) was used. Infected Vero E6 cells were fixed with 4% formaldehyde in phosphate buffer saline (PBS) for 1 hour at room temperature then washed twice and permeabilized with 0.5% Triton-X in PBS for 10 minutes. After 2x washes, mouse-anti-dsRNA antibody 1:200 in PBS was added and incubated for 90 minutes, followed by 3x washes and incubation with goat-anti-mouse Alexa^488^ (Jackson ImmunoResearch, USA, Code: 115-545-003) for 1 hour. Cells were washed x3, incubated with DAPI (5μg/mL, Invitrogen, Code: D1301) for 2 minutes, washed again ×3 and infected cells visualized with a Leica DMi8 microscope. All incubations were performed in room temperature and all washes were done with PBS.

**RNA extraction and real time PCR**

RNA extraction was done using QIAamp Viral RNA Mini Kit (Qiagen, Hilden, Germany) as per the manufacturer’s instructions, and reverse transcription was carried out using iScript™ cDNA Synthesis Kit (Biorad, Solna, Sweden). The real time PCR was performed on CFX96 (Biorad, Solna, Sweden) using iTaq Universal Probes Supermix (Biorad, Solna, Sweden) with following cycling conditions: initial denaturation at 95^o^C for 3 min, followed by 45 cycles of 95°C for 5 seconds and 60°C for 30 seconds. The primers (RdRp_SARSr-F and RdRp_SARSr-R) and probe (RdRp_SARSr-P2) targeting the RdRp gene of SARS coronavirus-2 that has been described previously (1). A 10-fold serial dilution of plasmid (pEX-A128-nCoV_RdRP, Eurofins Genomics, Germany), ranging from 2×10^8^ to 2×10^0^ copies was used for quantification.
